# Tumor Differentiation is the Dominant Prognostic Factor for Patients with Colorectal Neuroendocrine Neoplasms with Distant Metastasis

**DOI:** 10.1155/2022/1720624

**Published:** 2022-12-19

**Authors:** Zhijie Wang, Susheng Shi, Hongchang Ren, Qian Liu

**Affiliations:** ^1^Department of Colorectal Surgery, National Cancer Center, National Clinical Research Center for Cancer, Cancer Hospital, Chinese Academy of Medical Sciences and Peking Union Medical College, Beijing 100021, China; ^2^Department of Pathology, National Cancer Center, National Clinical Research Center for Cancer, Cancer Hospital, Chinese Academy of Medical Sciences and Peking Union Medical College, Beijing 100021, China; ^3^Department of General Surgery, Strategic Support Force Medical Center, Beijing 100101, China

## Abstract

**Purpose:**

Colorectal neuroendocrine neoplasms (NENs) are rare tumors. The prognosis and prognostic factors of metastatic colorectal NENs have not been fully elucidated.

**Methods:**

We retrospectively enrolled 77 consecutive patients diagnosed with colorectal NENs with synchronous distant metastases between 2000 and 2021. All patients were assigned to the neuroendocrine tumor (NET) group or the neuroendocrine carcinoma (NEC) group based on histological differentiation. Propensity score matching (PSM) was performed to minimize confounding bias. The Kaplan–Meier method was used to calculate the survival rates. Univariate and multivariate logistic regression analyses were performed to identify prognostic factors.

**Results:**

In total, 35 (45.5%) and 42 (54.5%) patients had well-differentiated NETs and poorly differentiated NECs, respectively. The median overall survival (OS) was 26 months for the entire cohort, and the 1-year, 3-year, and 5-year OS rates were 69.4%, 41.4%, and 27.8%, respectively. In the subgroup analysis, the median OS was 62 and 10 months for NETs and NECs, respectively. Univariate analysis demonstrated that patients with a primary tumor located in the colon, ulcerative tumors and poorly differentiated tumors were at higher risk for poorer progression-free survival (PFS) and OS. However, only histological differentiation was identified as an independent factor affecting OS (hazard ratio (HR) = 8.28, 95% confidence interval (CI): 2.98–23.01, *P* < 0.001) in multivariate analysis. After PSM, histological differentiation was further confirmed as the dominant factor affecting OS (HR = 6.09, 95% CI: 1.96–18.95, *P*=0.002)).

**Conclusion:**

Histological differentiation was the most dominant prognostic factor in patients with metastatic colorectal NENs. Patients with well-differentiated NETs had a good chance of long-term survival.

## 1. Introduction

Colorectal neuroendocrine neoplasms (NENs) are a rare subtype of colorectal tumors. However, a rapidly increasing incidence of colorectal NENs has been observed in recent decades, owing to the utilization of colonoscopy screening [1–3]. Colorectal NENs are a group of highly heterogeneous tumors with significantly different clinical features and outcomes based on their pathological manifestations [4]. According to the 2019 edition of the WHO classification system, colorectal NENs are categorized into G1, G2, and G3 NENs based on the Ki-67 index and mitotic count. All G1 and G2 NENs are regarded as well-differentiated and are named G1 and G2 neuroendocrine tumors (NETs), respectively. However, for G3 NENs, well-differentiated G3 NENs are termed G3 NETs, whereas poorly differentiated G3 NENs are termed neuroendocrine carcinomas (NECs) [5, 6].

Most colorectal NENs are small and early submucosal lesions, and endoscopic resection is a sufficient and reliable treatment option, guaranteeing a favorable prognosis [7]. Metastatic NENs are infrequent and constitute only 5.5%–14% of all colorectal NENs at the initial diagnosis [8–11]. However, the rates of distant metastasis vary widely from G1 to G3 NENs. For patients with G1 and G2 NENs, metastatic NENs are found in only 0.3% and 6.3% of these patients, respectively [12]. More than half of patients with G3 NENs show distant metastasis at the date of diagnosis [13, 14]. Metastatic G3 colorectal NENs are highly aggressive and malignant, with a median overall survival time of only 4–10 months [14, 15].

Owing to the rarity of metastatic colorectal NENs, there is still no widely acknowledged consensus regarding optimal treatment strategies. In addition, the prognostic factors for these patients have not been well understood in previous studies. In the present study, we aimed to evaluate the clinical outcomes of metastatic colorectal NENs and identify important prognostic factors that will aid in the management of these tumors.

## 2. Materials and Methods

### 2.1. Study Design and Participants

This study received approval from the Ethics Committee of the National Cancer Center and was performed in accordance with the Declaration of Helsinki of the World Medical Association. We conducted a retrospective study and enrolled 69 and 8 patients diagnosed with metastatic colorectal NENs between 2000 and 2021 from the National Cancer Center/Cancer Hospital, the Chinese Academy of Medical Sciences and the Strategic Support Force Medical Center, respectively. The inclusion criteria were as follows: (1) all cases were pathologically confirmed as colorectal NENs, and (2) all cases presented with synchronous metastatic disease at the initial diagnosis. The exclusion criteria were as follows: (1) patients suffering from another malignant tumor and (2) patients with incomplete clinicopathological data. Finally, a total of 77 eligible patients were included in the present study. We extracted clinicopathological information from the electronic medical database and obtained survival data through telephone calls or outpatient visits. The last date of follow-up was April 30, 2022.

The primary outcomes were the survival outcomes of these advanced patients, including progression-free survival (PFS) and overall survival (OS). The PFS was calculated between the initial anticancer treatment date and the tumor progression date based on imaging techniques. OS was calculated between the date of initial anticancer treatment and the date of cancer-specific death. The secondary outcomes were the prognostic factors that affected PFS and OS.

### 2.2. Statistical Analysis

**Figure 1:**
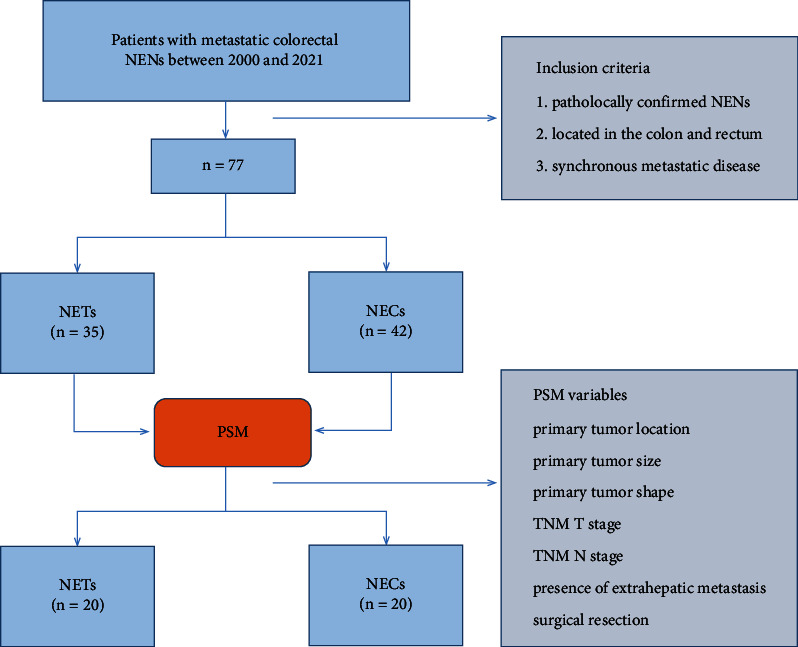
Flow chart presenting the patients' enrollment in our study.

Continuous data that followed a normal distribution were expressed as means and standard deviations (SDs), and then they were compared using a *t* test. Continuous data that did not follow a normal distribution are presented as medians and ranges, and they were compared using the Mann–Whitney *U* test. Qualitative and ordinal data are presented as counts and percentages, and they were compared using the *X*^2^ test or Fisher's exact test for categorical data and the Mann–Whitney *U* test for ordinal data. To reduce the imbalance between the NET group and the NEC group, propensity score matching (PSM) was performed by fitting a logistic regression model and setting the caliper at 0.05. One-to-one pair matching was performed without replacement, and 20 matched pairs were selected (Figure 1). The covariates included the primary tumor location, size, shape, TNM *T* stage, TNM N stage, presence of extrahepatic metastasis, and surgical treatment. PFS and OS were calculated using Kaplan–Meier curves and compared using the log-rank test. Univariate and multivariate logistic regression analyses were conducted to identify independent prognostic factors. All data calculations and analyses were performed using Statistical Package for the Social Sciences (SPSS version 24.0; IBM Corp., Armonk, NY). Statistical significance was set at a two-sided*P* value < 0.05.

## 3. Results

### 3.1. Clinicopathological Characteristics

The clinical and pathological data are shown in Table 1. From 2000 to 2021, a total of 77 consecutive patients were retrospectively included in this study with an average age of 57.7 ± 12.2 years. In total, 43 (55.8%) and 34 (44.2%) patients were males and females, respectively. Hematochezia (35.1%) and changes in bowel habits (35.1%) were the most common clinical manifestations, followed by abdominal pain (27.3%) and distention (10.4%). No cases of carcinoid syndrome were found. Remarkably, 9 (11.7%) patients were asymptomatic and diagnosed through routine physical examination. With regard to the location of the primary tumor, more than half (72.7%) of the patients had their primary tumor located in the rectum, followed by the ascending colon (10.4%) and sigmoid colon (7.8%). The liver was the most common organ of distant metastasis (80.5%), and 20 (26.0%) and 17 (22.1%) individuals presented with distant lymph node and bone metastases, respectively. Surprisingly, lung metastasis was less likely than expected, and only 1 (1.3%) patient suffered from lung metastasis.

With regard to the pathological features, 8 (10.4%), 19 (24.7%), 7 (9.1%), and 42 (54.5%) patients had G1 NETs, G2 NETs, G3 NETs, and G3 NECs, respectively. The median Ki-67 index was 40% (range 1%–95%). Regarding the macroscopic morphology of primary tumors, 37 (48.1%) and 40 (51.9%) primary tumors were ulcerative and protruding lesions, respectively. The immunohistochemical examination demonstrated that 74 (96.1%) and 68 (88.3%) patients were positive for synaptophysin and CD56 expression, respectively.

The treatment-modality data for these patients are shown in Table 1. A total of 35 (45.5%) individuals received surgical resection, including 12 (15.6%) individuals who underwent radical excision and 23 (29.9%) individuals who underwent palliative excision. Most (83.1%) of the cohort received systematic chemotherapy, and only 4 (5.2%) patients received radiotherapy. Four (5.2%) patients refused to receive any treatment.

### 3.2. Propensity Score Matching

The differences in the clinicopathological variables between patients with metastatic colorectal NETs or NECs before and after PSM are detailed in Table 2. In the original cohort, significant differences regarding primary tumor location, size, shape, invasion depth, and presence of extrahepatic metastasis were observed between NETs and NECs. After PSM, these factors were comparable between the NET group and the NEC group.

### 3.3. Oncological Outcomes

**Figure 2:**
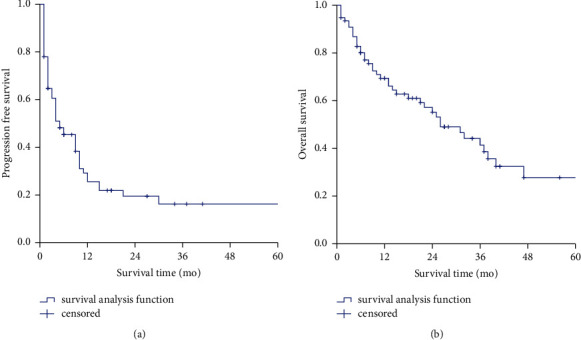
Kaplan–Meier survival analyses of metastatic colorectal NENs. (a) PFS of the whole cohort, (b) OS of the whole cohort.

A median follow-up duration of 15 (range 1–88) months was obtained in this study. Six patients were lost to follow-up due to a loss of communication, leading to a follow-up rate of 92.2%. The median PFS was only 5 months, and the 1-year, 3-year, and 5-year PFS rates were 25.5%, 16.2%, and 16.2%, respectively (Figure 2(a)). The median OS was 26 months, and the 1-year, 3-year, and 5-year OS rates were 69.4%, 41.4%, and 27.8%, respectively (Figure 2(b)).

The univariate and multivariate analyses of the risk factors affecting PFS and OS in the original cohort are presented in Table 3. We demonstrated that the histological differentiation (*P*=0.001), location (*P*=0.010), and macroscopic morphology (*P*=0.007) of the primary tumor were associated with PFS using univariate Cox proportional hazards regression analysis. However, none of these variables were confirmed as independent risk factors affecting PFS in subsequent multivariate analysis. Using univariate Cox proportional hazards regression analysis, we found that histological differentiation (*P* < 0.001), location (*P*=0.003), and macroscopic morphology (*P* < 0.001) of the primary tumor were associated with OS. Subsequent multivariate analysis indicated that tumor differentiation was the only prognostic factor that significantly affected OS ((hazard ratio (HR) = 8.28, 95% confidence interval (CI): 2.98–23.01, *P* < 0.001)).

**Figure 3:**
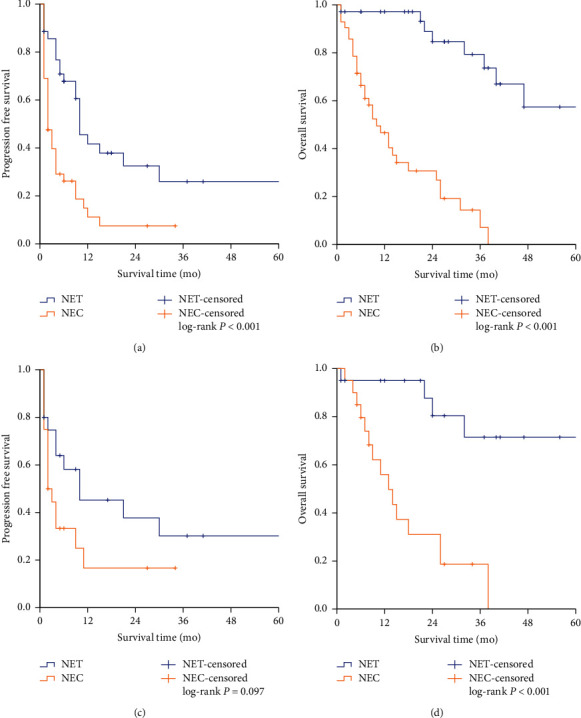
Kaplan–Meier survival analyses of metastatic colorectal NENs by histological differentiation before and after PSM. (a) PFS of NETs and NECs in the original cohort, (b) OS of NETs and NECs in the original cohort, (c) PFS of NETs and NECs in the matched cohort, (d) OS of NETs and NECs in the matched cohort.

For patients with colorectal NETs, the median PFS was 10 months, and the 1-year and 3-year PFS rates were 41.7% and 32.5%, respectively. However, for patients with colorectal NECs, the median PFS was only 2 months, and the 1-year and 3-year PFS rates were 11.2% and 7.5%, respectively (Figure 3(a)). Regarding OS, patients with colorectal NETs reached a satisfactory median OS of 62 months, and the 1-year and 3-year OS rates were 97.1% and 79.3%, respectively. In contrast, the patients with colorectal NECs had significantly decreased OS; the median OS was only 10 months, and the 1-year and 3-year OS rates were 46.6% and 30.8%, respectively (Figure 3(b)).

The univariate and multivariate analyses of the risk factors affecting PFS and OS in the matched cohort are presented in Table 4. No significant variables affecting PFS were found. Tumor differentiation was further confirmed as the only prognostic factor that significantly affected OS (HR = 6.09, 95% CI: 1.96–18.95, *P*=0.002)). The survival curves of PFS and OS in the matched cohorts are shown in Figure 3. There was no significant difference in PFS between NETs and NECs. However, the median OS was 88 months in the NET group and 13 months in the NEC group, and this difference was statistically significant (*P* < 0.001).

## 4. Discussion

Colorectal NENs are a group of rare tumors with high heterogeneity, and the vast majority of them are indolent, well-differentiated, and localized lesions at an early stage, which can be cured through endoscopic resection [16, 17]. Metastatic NENs represent only a small fraction of all colorectal NENs. In a study on 5457 patients with colorectal NENs from the National Cancer Database (NCDB), Chagpar et al. reported that only 299 (5.5%) patients demonstrated distant metastasis [8]. In another study on 607 patients with hindgut NENs, Kim et al. reported that only 42 (6.9%) patients presented extensive disease [9]. Although distant metastasis only occurs in a small portion of all colorectal NENs, the rates vary widely based on their grade and differentiation. In a study that included 9926 NENs of the colon and rectum from the Surveillance, Epidemiology, and End Results (SEER) database, Ding et al. found that only 1092 (11.0%) individuals suffered from metastatic disease; however, in a subgroup analysis, metastatic disease developed in 3.6% and 41.1% of patients with well-differentiated NETs and poorly differentiated NECs, respectively [10]. Another report from the C-NET study, a Japanese multicenter prospective study of colorectal NETs, included 500 colorectal NETs, and only 3 (0.6%) of them presented metastatic disease [18]. However, most previous studies have shown that more than half of colorectal NECs have distant metastases at the date of initial diagnosis. Owing to the rarity of metastatic colorectal NETs, few studies have described the prognosis of patients and compared the difference between NETs and NECs.

The underlying genetic and molecular mechanisms as well as the risk factors for distant metastasis have not been fully elucidated in previous studies, especially for colorectal NETs. Generally, NETs with a size of <1 cm are at an extremely low risk of metastasis and can be endoscopically resected with a favorable prognosis [19, 20]. However, in the present study, we observed 6 metastatic NETs with primary lesions smaller than 1 cm. Few previous studies have focused on metastatic NETs originating from diminutive primary tumors. One available study has reported that 1% of all colorectal NENs smaller than 1 cm are at risk of distant metastasis [21]. Another study has reported that 3.7% of all colorectal NENs smaller than 2 cm may develop metastatic disease [22]. It remains unknown why these diminutive and indolent NETs develop distant metastases, thus requiring further exploration. However, these results may indicate that even for small and localized NETs, it is necessary to evaluate patients with whole-body imaging to exclude metastatic disease before endoscopic therapy, which may be missed when following current guidelines in clinical practice [17, 23].

The present cohort had a median OS of 26 months with 3-year and 5-year OS rates of 41.4% and 27.8%, respectively. In a real-world data retrospective cohort study from the SEER database with 9732 patients with metastatic colorectal tumors, Han et al. reported that 80.95% of these patients had colorectal adenocarcinomas; they reported a median OS of 23 months, and the 3-year and 5-year OS rates were 32.4% and 18.4%, respectively [24]. Compared to the overall population of people with colorectal cancer, colorectal NENs may have a higher probability of long-term survival. However, colorectal NENs are a group of highly heterogeneous tumors. In the present study, univariate analysis indicated that metastatic NENs with a protruding and well-differentiated primary tumor located in the rectum were prone to have significantly better PFS and OS than metastatic NENs with an ulcerative and poorly differentiated primary tumor located in the colon.

In the present study, only histological differentiation was confirmed as an independent prognostic factor that affected OS. Although all patients had distant metastases, there was a significantly favorable prognosis for NETs compared to NECs. Patients with metastatic NETs reached a satisfactory median OS of 62 months, and the 1-year and 3-year OS rates were 97.1% and 79.3%, respectively. There are few data regarding the survival outcomes of metastatic colorectal NETs in previous literature. Kong et al. reported a median OS of 87 months in 27 patients with metastatic rectal NENs; 20 (74.1%) of these patients were determined to have well-differentiated NETs, and 7 (25.9%) of these patients were uncertain. These data, together with the present data, suggest that patients with colorectal NETs may achieve long-term survival despite the presence of distant metastases. However, patients with metastatic NECs showed an extremely dismal median OS of 10 months in the present study, with 1-year and 3-year OS rates of 46.6% and 30.8%, respectively. These results agreed with several previous studies, which reported a median OS of 4–10 months [14, 15]. The significant differences in survival outcomes indicated that accurate determination of tumor differentiation is crucial in predicting prognosis. However, assessing the degree of tumor differentiation is challenging. In earlier studies, all G1 and G2 NENs were regarded as well-differentiated NENs, while all G3 NENs were acknowledged as poorly differentiated NECs, which was based on the World Health Organization (WHO) 2010 classification and nomenclature system for digestive NENs [17]. Recent studies have described a new entity of high-grade, well-differentiated NENs (NET G3) and separated them from the prior G3 NENs [25]. In the recent 2019 edition of the WHO classification system, G1, G2, and G3 well-differentiated NENs are named NETs, while G3 poorly differentiated NENs are termed NECs [5, 6]. However, it is challenging to distinguish G3 NETs from NECs in many cases based only on morphology differentiation. Genetic information and proliferative activity are often taken into account. NENs with mutations in KRAS, BRAF, p53, and Rb1 or with a Ki-67 index greater than 70%–80% are usually diagnosed as NECs [26, 27].

There are limited reports regarding the genetic discrepancies between colorectal NETs and NECs. Chen et al. extracted data from the American Association for Cancer Research (AACR) Project Genomics, Evidence, Neoplasia, Information, and Exchange (GENIE) database and accessed genetic data from 83 colorectal NECs, 276 gastrointestinal NETs, and 6476 colorectal adenocarcinomas. These authors demonstrated that colorectal NECs presented a significantly higher tumor mutation burden (TMB) (5.16 versus 1.43) and a higher somatic mutation rate of TP53 (65.5% versus 6.8%) than colorectal NETs. With regard to copy number alteration (CNA), colorectal NECs usually had CNA frequently in chromosomes 8, 9, 13, 16, 17, and 20 with a gain of MYC (12.3%), loss of RB1 (10.7%), and loss of PTEN (5.4%) being the most common CNAs, while colorectal NETs often had the most CNAs occurring in chromosomes 3, 9, 11, 12, 17, and 18 with a low CNA frequency [28]. Chen et al. concluded that colorectal NECs are more similar to colorectal adenocarcinomas. The genetic difference between colorectal NETs and NECs may explain their significantly different clinical outcomes and further guide individualized treatment for these advanced patients.

The present study had aforementioned limitations. Due to the retrospective nature of the present study, bias arising from the collection of information could not be completely avoided. Further prospective studies are needed to delineate the clinicopathological and genetic characteristics of NENs as well as the clinical outcomes of patients. The second limitation was the small sample size of the present study. Owing to the rarity of colorectal NENs and the low proportion of metastatic disease, we enrolled a limited number of patients over a period of 20 years. Multicenter studies from more hospitals should be performed to include more patients in further studies.

## 5. Conclusion

Metastatic colorectal NENs are a group of highly heterogeneous tumors, and histological differentiation is the most important prognostic factor. Metastatic patients with well-differentiated colorectal NETs have a favorable prognosis, while those with poorly differentiated colorectal NECs have poor survival outcomes.

## Figures and Tables

**Table 1:** Clinical and pathological features.

Characteristic	Patients (*n* = 77)
Sex, *n* (%)	
Male	43 (55.8%)
Female	34 (44.2%)
Age [years, mean ± SD]	57.7 ± 12.2
BMI (kg/m^2^, mean ± SD)	23.7 ± 3.4
Symptoms, *n* (%)	
Hematochezia	27 (35.1%)
Changes in bowel habits	27 (35.1%)
Abdominal pain	21 (27.3%)
Abdominal distention	8 (10.4%)
Obstruction	7 (9.1%)
Weight loss	3 (3.9%)
Anemia	2 (2.6%)
Carcinoid syndrome	0
Asymptomatic	9 (11.7%)
Primary sites, *n* (%)	
Rectum	56 (72.7%)
Sigmoid	6 (7.8%)
Descending colon	1 (1.3%)
Transverse colon	2 (2.6%)
Ascending colon	8 (10.4%)
Cecum	4 (5.2%)
Primary tumor size (median (range), cm)	4.0 (0.4–15.0)
Primary tumor size, *n* (%)	
≤1 cm	6 (7.8%)
1-2 cm	11 (14.3%)
>2 cm	60 (77.9%)
Primary tumor shape, *n* (%)	
Ulcerative type	37 (48.1%)
Protrude type	40 (51.9%)
TNM T stage, n (%)	
T1	8 (10.4%)
T2	3 (3.9%)
T3	37 (48.1%)
T4	29 (37.7%)
TNM N stage, *n* (%)	
N0	9 (11.7%)
N1	68 (88.3%)
Sites of distant metastases, *n* (%)	
Liver	62 (80.5%)
Liver only	33 (42.9%)
Distant lymph nodes	20 (26.0%)
Bone	17 (22.1%)
Peritoneum	8 (10.4%)
Lung	1 (1.3%)
Adrenal	1 (1.3%)
Pancreas	1 (1.3%)
Grade and differentiation	
G1 NET	9 (11.7%)
G2 NET	19 (24.7%)
G3 NET	7 (9.1%)
G3 NEC	42 (54.5%)
Ki 67 (median, range)	40% (1%–95%)
Synaptophysin, *n* (%)	
Positive	74 (96.1%)
Negative	3 (3.9%)
CD56, *n* (%)	
Positive	68 (88.3%)
Negative	9 (11.7%)
Surgery, *n* (%)	35 (45.5%)
Radical resection	12 (15.6%)
Palliative resection	23 (29.9%)
Systematic chemotherapy, *n* (%)	64 (83.1%)
Radiotherapy, *n* (%)	4 (5.2%)
No treatment, *n* (%)	4 (5.2%)

SD, standard deviation; BMI, body mass index; NET, neuroendocrine tumor; NEC, neuroendocrine carcinoma.

**Table 2:** Clinicopathological data between patients with metastatic colorectal NETs and NECs before and after propensity score matching.

Characteristic	Original cohort			Matched cohort		
	NET (*n* = 35)	NEC (*n* = 42)	*P*	NET (*n* = 20)	NEC (*n* = 20)	*P*
Sex, *n* (%)			0.802			0.744
Male	19 (54.3%)	24 (57.1%)		13 (65.0%)	12 (60.0%)	
Female	16 (45.7%)	18 (42.9%)		7 (35.0%)	8 (40.0%)	
Age (years, mean ± SD)	57.1 ± 2.0	58.9 ± 2.0	0.609	57.5 ± 2.7	58.97 ± 2.7	0.757
BMI (kg/m^2^, mean ± SD)	24.3 ± 0.7	23.2 ± 0.5	0.189	23.5 ± 0.8	23.8 ± 0.6	0.828
Primary tumor location, *n* (%)			<0.001			1.000
Rectum	32 (91.4%)	23 (54.8%)		17 (85.0%)	18 (90.0%)	
Colon	3 (8.6%)	19 (45.2%)		3 (15.0%)	2 (10.0%)	
Primary tumor size, *n* (%)			0.008			1.000
≤2 cm	12 (34.3%)	4 (9.5%)		3 (15.0%)	3 (15.0%)	
>2 cm	23 (65.7%)	38 (90.5%)		17 (85.0%)	17 (85.0%)	
Primary tumor shape, (%)			0.001			0.752
Ulcerative type	24 (68.6%)	13 (31.0%)		10 (50.0%)	9 (45.0%)	
Protrude type	11 (31.4%)	29 (69.0%)		10 (50.0%)	11 (55.0%)	
TNM T stage, *n* (%)			0.009			1.000
T1 and T2	9 (25.7%)	2 (4.8%)		1 (5.0%)	1 (5.0%)	
T3 and T4	26 (74.3%)	40 (95.2%)		19 (95.0%)	19 (95.0%)	
TNM N stage, *n* (%)			0.771			1.000
N0	5 (14.3%)	4 (9.5%)		2 (10.0%)	2 (10.0%)	
N1	30 (85.7%)	38 (90.5%)		18 (90.0%)	18 (90.0%)	
Extrahepatic metastasis, *n* (%)			0.021			0.744
Yes	15 (42.9%)	29 (69.0%)		8 (40.0%)	7 (35.0%)	
No	20 (57.1%)	13 (31.0%)		12 (60.0%)	13 (65.0%)	
Synaptophysin, *n* (%)			0.872			1.000
Positive	33 (94.3%)	41 (97.6%)		19 (95.0%)	20 (100%)	
Negative	2 (5.7%)	1 (2.4%)		1 (5.0%)	0 (0)	
CD56, *n* (%)			0.065			1.000
Positive	34 (97.1%)	34 (81.0%)		19 (95.0%)	18 (90.0%)	
Negative	1 (2.9%)	8 (19.0%)		1 (5.0%)	2 (10.0%)	
Surgical resection, *n* (%)	19 (54.3%)	16 (38.1%)	0.155	11 (55.0%)	10 (50.0%)	0.752

NET, neuroendocrine tumor; NEC, neuroendocrine carcinoma; SD, standard deviation; BMI, body mass index.

**Table 3:** Univariate and multivariate analysis of the risk factors of PFS and OS in the original cohort.

Variables	PFS				OS			
	Univariate analysis		Multivariate analysis		Univariate analysis		Multivariate analysis	
	HR (95% CI)	*P*	HR (95% CI)	*P*	HR (95% CI)	*P*	HR (95% CI)	*P*
Sex (male/female)	1.30 (0.76–2.21)	0.335			1.08 (0.58–2.03)	0.802		
Age	1.00 (0.97–1.02)	0.504			1.01 (0.98–1.04)	0.511		
BMI	0.98 (0.91–1.05)	0.509			1.00 (0.92–1.09)	0.926		
Primary tumor location (colon/rectum)	2.10 (1.19–3.68)	0.010	1.68 (0.93–3.05)	0.086	2.74 (1.42–5.29)	0.003	0.74 (0.37–1.47)	0.392
Primary tumor size (>2 cm/≤ 2 cm)	2.00 (0.97–4.10)	0.060			2.00 (0.83–4.82)	0.122		
Primary tumor shape (ulcerative/protruding)	2.16 (1.24–3.76)	0.007	1.75 (0.95–3.21)	0.073	3.57 (1.79–7.13)	<0.001	1.44 (0.67–3.09)	0.349
Tumor differentiation (NEC/NET)	2.64 (1.51–4.63)	0.001	1.37 (0.71–2.63)	0.347	10.97 (4.37–27.54)	<0.001	8.28 (2.98–23.01)	<0.001
TNM *T* stage (T3 and T4/T1 and T2)	1.15 (0.54–2.44)	0.714			2.47 (0.76–8.03)	0.132		
TNM N stage (N1/N0)	0.70 (0.33–1.48)	0.349			2.42 (0.72–8.20)	0.155		
Extrahepatic metastasis (Yes/No)	1.09 (0.64–1.86)	0.750			1.21 (0.65–2.26)	0.547		
Surgical resection (Yes/No)	0.99 (0.58–1.67)	0.955			1.33 (0.71–2.48)	0.376		

PFS, progression-free survival; OS, overall survival; HR, hazard ratio; CI, confidence interval; BMI, body mass index; NET, neuroendocrine tumor; NEC, neuroendocrine carcinoma.

**Table 4:** Univariate and multivariate analysis of the risk factors of PFS and OS in the matched cohort.

Variables	PFS				OS			
	Univariate analysis		Multivariate analysis		Univariate analysis		Multivariate analysis	
	HR (95% CI)	*P*	HR (95% CI)	*P*	HR (95% CI)	*P*	HR (95% CI)	*P*
Sex (male/female)	1.61 (0.72–3.60)	0.251			1.21 (0.48–3.05)	0.691		
Age	0.99 (0.96–1.02)	0.525			1.00 (0.95–1.04)	0.831		
BMI	1.04 (0.93–1.17)	0.477			1.10 (0.95–1.26)	0.202		
Primary tumor location (colon/rectum)	1.26 (0.38–4.24)	0.707			0.52 (0.07–3.87)	0.519		
Primary tumor size (>2 cm/≤ 2 cm)	1.17 (0.40–3.41)	0.774			1.77 (0.41–7.68)	0.449		
Primary tumor shape (ulcerative/protruding)	1.12 (0.79–1.61)	0.525			2.80 (1.05–7.46)	0.039	2.10 (0.79–5.62)	0.139
Tumor differentiation (NEC/NET)	1.83 (0.84–3.99)	0.129			7.08 (2.28–22.01)	0.001	6.09 (1.96–18.95)	0.002
TNM *T* stage (T3 and T4/T1 and T2)	0.37 (0.05–3.00)	0.350			0.20 (0.02–1.68)	0.139		
TNM N stage (N1/N0)	0.62 (0.22–1.81)	0.383			2.40 (0.32–18.12)	0.396		
Extrahepatic metastasis (Yes/No)	0.69 (0.32–1.51)	0.354			0.76 (0.30–1.88)	0.548		
Surgical resection (Yes/No)	1.13 (0.53–2.40)	0.761			1.71 (0.66–4.43)	0.268		

PFS, progression-free survival; OS, overall survival; HR, hazard ratio; CI, confidence interval; BMI, body mass index; NET, neuroendocrine tumor; NEC, neuroendocrine carcinoma.

## Data Availability

The data used to support the findings of this study are available from the corresponding author upon request.
